# Reply on Behalf of the Class of 1874

**Published:** 1874-05

**Authors:** Geo. H. Mosher


### REPLY ON BEHALF OF THE CLASS OF 1874.

BY GEO. H. MOSHER.

*Respected Sirs* :—This is a day to which I doubt not, you as instructors, and certainly we as students, have been for nearly half a year anxiously and hopefully looking ; to us a day of mingled joys and regrets, of regrets of the necessity of separation from the tried and true friends which we have gained in you, of joys at the prospect of rejoining at our homes the loved ones from whom we have been so long separated and of other joys, that we are now to take a place in the bustling, teeming world of business and professional life, and evince by our works the results of our instructions in this honored institution. Therefore, we thank you most heartily for the diplomas, for the valuable advice and good wishes, and for these volumes of holy writ, which we have just received at your hands. We trust that our professional lives may never discredit our diplomas, that we shall never neglect to heed the good advice, and never forget the kind wishes of our instructors, and that the sacred truths of the book of books may sink deep in our hearts, and be of abiding and everlasting benefit to each one of the class. While we would not in the slightest degree detract from, or in any manner undervalue our diplomas, still, we are sensible that pieces of parchment do not make a graduate a successful professional man, and that if we are to attain professional eminence, it must be by continued and persistent labor, nor must we think that because we have graduated from this honored institution our student life is finished and our education complete. No ! these diplomas are simply evidence that we are now ready to commence the battle of professional life, our student life must continue, our education is never complete. Whatever may have been our opinions, when we each determined to make dentistry our profession, we are now convinced that nothing but faithful, continued, and well directed effort will accomplish the professioual perfection, to which our earliest aspirations pointed. We esteem the instruction received at your hands since we have been in attendance upon this institution, in the light of a wisley built and solid foundation, upon which we are to erect a structure, which shall be success, the chief component parts of which are fame, fortune and friends. That each of us will build that structure, we, each for himself, is no doubt morally certain, and that we have your best wishes, in so building, we have too many evidences to indulge in a doubt. We regard the knowledge gained by us while here, as eminently practical and useful, and it gives us great pleasure to say, that our time and money spent in pursuing our studies has been most favorably invested, and if we are half as faithful in the exercise of our professional knowledge and skill as you have been in imparting to us the results of your life long studies and experience, we shall attain such success in life as shall lead us to a continued thankfulness that we placed ourselves under your instruction. Sirs, may your efforts to disseminate useful knowledge, and your endeavors to raise the standard of our honored profession meet with the success to which your proudest hopes have reached, and that your lives may be even more prosperous and your old age abundantly honored, is the earnest wish and prayer of the class of 1874.

*Fellow Classmates*:—When I reflect that our instructors and other eminent men in our chosen profession had so few advantages when they commenced the study of dentistry, that, thirty years ago, there was almost no such a thing as dental literature; that dental colleges were not even dreamed of; that they as students were groping in the darkness, and could hope to gain little knowledge except that which they might be enabled to study out by themselves, and when I see the wonderful progress which has been made, the abundance of dental literature, our numerous dental colleges, and the many advantages now afforded the student for attaining a thorough knowledge of dentistry in all its branches, and realize the change from the midnight darkness of those other days to the bright noon-day of the present; I can but feel the greatness of the duty which we owe to our God, the world and to ourselves, and of the importance of our aid to keep the car of progress rolling onward, and, if we would not be left behind by the quick succession of events, we must work valiantly and faithfully for the best interests of our noble profession, keeping ourselves thoroughly posted in the current writings of others, liberally supporting our dental associations, and by patient investigation and experiments in our several laboratories we may hope not only to stand well among our own brethren in the profession, but we may be able to develop new truths which are as yet to the world unknown. Let us remember that we have chosen a profession in which what is called luck has no place, our success must be gained by persistent hard work, with heart, head and hands, and there is no doubt but that with such application, and the necessary adjunct of good health we may, any of us, expect to obtain just the success for which we hope.

Let us then be up and doing,
With a heart for any fate,
Still pursuing, still enduring;
Learn to labor, and to wait.

*Fellow Students*:—In one year more, you will stand in the places now occupied by us, one year more of study and patient application, and you will be prepared to take a place in the professional world as co-workers with your predecessors. If during your student life which precedes that day you shall have wasted any time, or in any manner misapplied your time and opportunities, you will sincerely regret it, for these are opportunities which to most men, occur but once in a lifetime, and it behooves you to improve every moment in a systematic endeavor to gain useful knowledge in order to commence your practice with the greatest possible advantage.

Here, and now we are to separate, teachers and pupils, each going his own way out into the world, and with some of us no doubt this separation is final, but however widely, and wherever our fortunes may lead us, we can all look back with pleasant recollections to the friendships and benfits gained while here, and with the fondest memories of our *Alma Mater*.

